# Fellow-Workers and the Roll of Honour

**Published:** 1916-01-29

**Authors:** 


### ROUND THE WORLD.

[The Editor invites Early Information and Contributions Marked "Fellow-Workers."]

### Fellow-Workers and the Roll of Honour.

Lieutenant W. W. Deans, R.A.M.C., who has died of wounds, obtained the L.R.C.P., L.R.C.S. diplomas at Edinburgh fifteen years ago.

Captain Edward A. Attwood, V.D., R.F.A., Special Reserve, better known to our readers as the Secretary of the London Homœopathic Hospital, has been with his brigade in action in the Ypres salient for the last few months, and has been mentioned in dispatches by Field-Marshal Sir John French for "gallant and distinguished conduct in the field."

Major J. D. Richmond, M.D., R.A.M.C., who was recently mentioned in General French's dispatch, was gazetted lieutenant in 1903. Before going to the Front he occupied the position of Deputy-Assistant Director of Medical Services, Scottish Command. He was formerly senior medical officer to the Walton Workhouse and Infirmary, Liverpool, and also held a resident appointment at the Birmingham City Hospital.

Lieutenant-Colonel W. Ranson, R.A.M.C., attached 3rd Northumbrian Field Ambulance (T.F.), who has been wounded, was formerly officer in charge of the Military Hospital, Beverley. He received his medical education at Edinburgh University and St. Thomas's Hospital, London, and became F.R.C.S. (Edin.) in 1898. Before the outbreak of war he was medical officer to the Beverley Cottage Hospital and medical officer of health to the Beverley Rural District Council, and at one time he held a resident appointment at Down County Infirmary, Downpatrick.

Captain F. A. Bearn, R.A.M.C., to whom a Military Cross has been awarded, is well known as a former house surgeon and house physician at the Manchester Royal Infirmary. Captain Bearn was wounded at Loos, and was mentioned in Sir John French's last dispatch. He received his medical training at Manchester, and obtained his medical and surgical degrees in 1913.

Captain Alexander Findlater, M.D., 1st London Mounted Brigade Field Ambulance, Royal Army Medical Corps, Territorial Force, has been appointed a Companion of the Distinguished Service Order for conspicuous gallantry and devotion to duty on several occasions, notably on September 29, 1915, at Chocolate Hill, Gallipoli Peninsula. He crossed over 200 yards of open ground under very heavy shell fire to render aid to two wounded men. He saved the life of one, but the other was beyond help. Dr. Findlater has practised at Edgware for many years, where he is well known and has hosts of friends. He obtained the M.D. degree at Dublin University in 1886.

Among those who have been awarded the Distinguished Conduct Medal for acts of gallantry and devotion to duty, and whose names appear in a Supplement to the *London Gazette* issued on January 23, are two ambulance workers: Corporal W. G. Muir, 1st/4th London Mounted Brigade Field Ambulance, R.A.M.C., Territorial Force, has received the award for conspicuous gallantry and devotion to duty on several occasions, notably at Chocolate Hill, Gallipoli Peninsula, on September 29, 1915, when he crossed over 200 yards of open ground under very heavy fire to assist the medical officer —presumably Dr. Findlater—to render aid to two wounded men. Private J. S. Kerr, R.A.M.C. (attached 7th Siege Battery, Royal Garrison Artillery), receives the medal for conspicuous gallantry and devotion to duty on December 29, 1915, near Ypres. During a heavy bombardment by high-explosive and gas shells he left his dug-out and passed through a dense cloud of gas over 200 yards to a farm in which another battery was billeted. Here he rendered first-aid to several wounded men. The farm was being heavily shelled at the time, but, though wounded himself, he continued his work among the other wounded.

Captain H. S. Milne, M.D., R.A.M.C., who has been wounded, obtained his medical and surgical degrees at Aberdeen in 1909.

Lieutenant M. A. Swan, M.B., R.A.M.C., attached R.F.A., who has been wounded while on service in the North of France, obtained his M.B. degree at Edinburgh University in 1901. He was for some time house surgeon and house physician at Alexandra Infirmary, Paisley, and when war broke out he was in practice at South Shields.

Lieutenant F. G. Foster, M.B., R.A.M.C., who is reported wounded, obtained his degree at Edinburgh University in 1913, and was subsequently house physician at the Royal Infirmary, Edinburgh.

The name of Captain B. C. A. Pockley, R.A.M.C., appears in the list of those officers who have received commendation for services during the daring raid on the wireless station at Bita Paka, in German New Guinea, during the early days of the war, on which occasion Captain Pockley was killed in action.

Captain J. W. Wood, R.A.M.C., attached 7th Seaforth Highlanders (T.F.), who is reported wounded, obtained the M.B. degree of Aberdeen University in 1914.

Lieutenant A. C. Edwards, M.B., R.A.M.C., attached King's Own (Yorks L.I.), 4th Battalion (T.F.), has been wounded. He obtained his medical training at Liverpool, and was formerly senior resident medical officer at the Northern Hospital, Liverpool.

Captain T. W. Wylie, M.B., R.A.M.C., who has been wounded, took his degree at Glasgow University in 1914.

Lieutenant H. Hogben, who was a student at Guy's Hospital when war broke out, and who was killed in action in the Persian Gulf a short time ago, is the subject of an appreciation in the current issue of *Guy's Hospital Journal*, in which are also printed two letters written by Lieutenant Hogben a short time before he fell. In these he vividly described some of the hardships of a campaign of which we have heard very little. He entered Guy's in October 1909, winning the London University Open Scholarship. At the outbreak of war he was working for his final examination, but as he already held a commission in the 10th Middlesex, he had to forgo his studies and answer to the mobilisation order.

Captain E. M. Skinner and Lieutenant N. S. Joshi, who have been wounded in the Persian Gulf, are officers in the Indian Medical Service.

